# Senolytic Treatment Improves Responsiveness to Mechanical Loading in the Skeleton of Aged Mice

**DOI:** 10.3390/ijms262211233

**Published:** 2025-11-20

**Authors:** Connor J. Cunningham, Hui Jean Kok, Joshua N. Farr, Sundeep Khosla, Alexander G. Robling

**Affiliations:** 1 Department of Anatomy, Cell Biology & Physiology, Indiana University School of Medicine, Indianapolis, IN 46202, USA; conjcunn@iu.edu (C.J.C.); jeankok@iu.edu (H.J.K.); 2 Division of Endocrinology, Mayo Clinic, Rochester, MN 55905, USA; farr.joshua@mayo.edu (J.N.F.); khosla.sundeep@mayo.edu (S.K.); 3 Robert and Arlene Kogod Center on Aging, Mayo Clinic, Rochester, MN 55905, USA; 4 Richard L. Roudebush Veterans Affairs Medical Center, Indianapolis, IN 46202, USA

**Keywords:** osteoporosis, aging, senolytics, Dasatinib, Quercetin, mechanotransduction

## Abstract

Aging plays a major role in the development of numerous chronic diseases, one of which is a marked decline in skeletal health. Beyond diminishing bone mass and strength, mammals of advanced age experience a decline in skeletal mechanotransduction—that is, the ability of the skeleton to respond adaptively to mechanical perturbation. One possibility for the loss of mechanotransduction in bone with aging is an age-associated increase in the population density of senescent cells—those cells that have undergone irreversible cell cycle arrest, resistance to apoptosis, and production of a modified secretome (the SASP) that has damaging effects to nearby healthy (non-senescent) cells. We investigated whether the presence of senescent cells might drive some of the diminished mechanical response observed in aged bone, by testing the hypothesis that the clearance of senescent cells via intermittent senolytic treatment promotes mechanical responsiveness in an aged skeleton. C57BL/6 mice aged 6 months and 22 months were treated weekly with the senolytic cocktail Dasatinib and Quercetin (D + Q) for 1 month, then subjected to low level in vivo mechanical loading of the ulna for 1 week. The 6-month-old mice exhibited a doubling of load-induced ulnar periosteal bone formation when treated with D + Q, compared to vehicle-treated mice, but the periosteal response to loading was not significantly altered by D + Q in the aged (22-month) mice. We further probed the efficacy of D + Q in mechanotransduction by switching to an endocortical model—the axial tibia loading system. Here, the 22-month-old mice had nearly double the load-induced endocortical bone formation compared to vehicle-treated mice. We further assayed the cortical bone gene expression profile in loaded and control tibias from treatment-naïve 6-month and 22-month mice, to determine whether there is significant overlap between mechanically induced signaling genes and SASP genes. We found significant load-induced changes among several SASP genes, suggesting that inhibition of the SASP (i.e., senomorphics) might interfere with mechanical signaling from otherwise healthy cells. In summary, clearance of senescent cells via intermittent D + Q treatment is effective at improving endocortical mechanical responsiveness in the aged skeleton, which is commonly diminished throughout the course of aging.

## 1. Introduction

With aging, the risk of developing numerous chronic diseases increases significantly [1]. Many of the underlying biological mechanisms that lead to age-associated chronic disease are not well known and likely vary by tissue type. In many tissues, the natural course of aging is associated with increased accumulation of senescent cells [2,3,4]. Biological senescence is defined as a cellular state of irreversible cell cycle arrest, resistance to apoptosis (the activation of the so-called senescent cell anti-apoptotic pathways, or SCAPs) and the initiation of a specific and caustic secretome, among other changes [5]. The modified secretome, known as the senescence-associated secretory phenotype (SASP), comprises the release of a collection of pro-inflammatory factors (e.g., IL-6 and TNF-α), matrix degraders (MMP1 and MMP3), and other cytokines (CCL2 and CCL20) that cause dysfunction in the otherwise healthy surrounding cells and tissues [6]. The accumulation of senescent cells within tissues establishes an environment of chronic inflammation, which over time, promotes the development of chronic disease states such as diabetes, cancer, and musculoskeletal degenerative disorders [7,8,9,10,11,12]. Senescence occurs in skeletal cells with aging [13].

Senescence in bone is characterized by, among other things, an increase in p16^INK4A^ expression, which is elevated throughout the course of aging [2]. p16^INK4A^ acts as a tumor suppressor and cell cycle regulator through the inhibition of CDK4 and CDK6 activity, preventing the downstream phosphorylation of retinoblastoma (Rb) protein [14]. In osteoprogenitor cells, the increase in p16^INK4A^ levels is associated with DNA damage and the development of the SASP [2]. One specific progeny of the osteoprogenitor cell, the osteocyte, has been a primary focus of skeletal senescence investigations due to its well-documented upregulation of a large number of SASP factors during aging [15].

To counteract or treat the deleterious effects of senescence in various cell types, there are currently two general biological mechanisms that can be targeted to counter those effects [16]. The first involves disarming the senescent cells’ strong anti-apoptotic program, so that the senescent cells can be induced to die and essentially be “cleared out” of the tissue. Agents that work via this mechanism are known as senolytics, because they target the senescent cell anti-apoptotic pathway (SCAP) and restore the cells’ ability to die [17]. A commonly used senolytic therapy comprises co-treatment with the FDA-approved tyrosine kinase inhibitor Dasatinib and the naturally occurring flavonoid Quercetin (D + Q) [18]. D + Q treatment inhibits BCL-2 family members to restore apoptosis potential [19]. Senolytics are the first subclass of senotherapeutics that have been successfully used to counteract the effects of senescent cells with aging, by disabling the senescent cells’ unique SCAP mechanism and promoting apoptosis of the cell with no off-target effects observed [20]. Senomorphics are a second subclass of senotherapeutics, and they work by inhibiting the release of SASP factors without causing cell death. The inhibition of SASP factors serves to protect nearby healthy cells and tissues from those inflammatory signals [20]. While senomorphics are effective at targeting the SASP, their utility in bone tissue has not been elucidated to the same degree as senolytics. One concern is that senomorphics inhibit the release of inflammatory factors from all cells (including healthy, non-senescent cells), and several key “healthy” processes in bone, like mechanotransduction, rely on a variety of inflammatory factors for signaling, albeit in smaller, more controlled amounts and distributions. Senomorphic-induced disruption of messenger signaling from healthy cells might therefore interfere with bone adaptation and the positive effects of exercise that originate from healthy (non-senescent) skeletal cells [21].

The skeletal response to mechanical stimulation is a crucial phenomenon for proper embryologic development, mineral accumulation, and postnatal skeletal function and integrity [22]. The process of mechanotransduction allows our skeletal elements to adapt to the prevailing load conditions, both positively (overuse) and negatively (disuse), and keeps bone mass, size, and strength in tune with mechanical inputs. This process has the ultimate effect of keeping skeletal elements healthy and fracture resistant [23]. Unfortunately, as we age, bone tissue’s ability to respond to osteogenic stimuli wanes significantly, to the point where our senior years are accompanied by an impaired response to load. The effect of aging on mechanical signaling in bone has been demonstrated in a number of animal models [24,25,26,27] and is observed clinically for older humans engaged in exercise studies [28,29,30,31]. There are many factors that could explain the loss of mechanosensitivity and mechanoresponsiveness in the aged skeleton, but one prominent mechanism that is gaining much attention is the skeletally potent phenomenon of cellular senescence [32,33]. Osteocytes are the main mechanoreception and signaling cells in bone, and the process of mechanotransduction in bone relies on a healthy, functional osteocyte population [21,34,35]. As the skeleton ages, osteocytes take on a senescent phenotype at an accelerated rate [2,36]. For example, senescent osteocytes comprise <3% of the cortical osteocyte population in 6 mo. mice, but their proportion rises to ~10% in 24 mo. mice, as assessed by DNA damage at the telomeres [37]. While the observed age-associated increase in senescent osteocytes does not appear overly dramatic (~7% increase), it represents a significant and disproportionate change in the osteocyte microenvironment [15]. A small number of senescent cells can have a large impact on neighboring healthy cells by “polluting” the local environment (the so-called “bystander effect”) with a multitude of detrimental secreted factors such as inflammatory cytokines, chemokines, proteases, and growth factors. SASP-enriched media from senescence-induced MLO-Y4 and MC3T3 cultures significantly suppresses mechanotransduction responses in naïve MLO-Y4 and MC3T3 cells subjected to fluid shear, suggesting that senescence/SASP mechanisms inhibit mechanotransduction in bone cells [38]. In the intact animal, osteocytes undergo senescence with increased age [39]. Collectively, those observations provided the rationale to investigate whether the biological process of senescence is at least partially responsible for deficiencies in mechanotransduction that occur with aging. We hypothesize that D + Q senolytic drug treatment is effective at removing senescent bone cells and can enhance load-induced bone formation within the aged skeleton. We addressed this hypothesis by conducting in vivo skeletal loading experiments in adult and aged mice exposed to senolytic treatment and measuring the response.

## 2. Results

### 2.1. Validation of Biological Activity of Senolytic Treatment: Effects on Bone Mass and Senescence Markers

**Figure 1:**
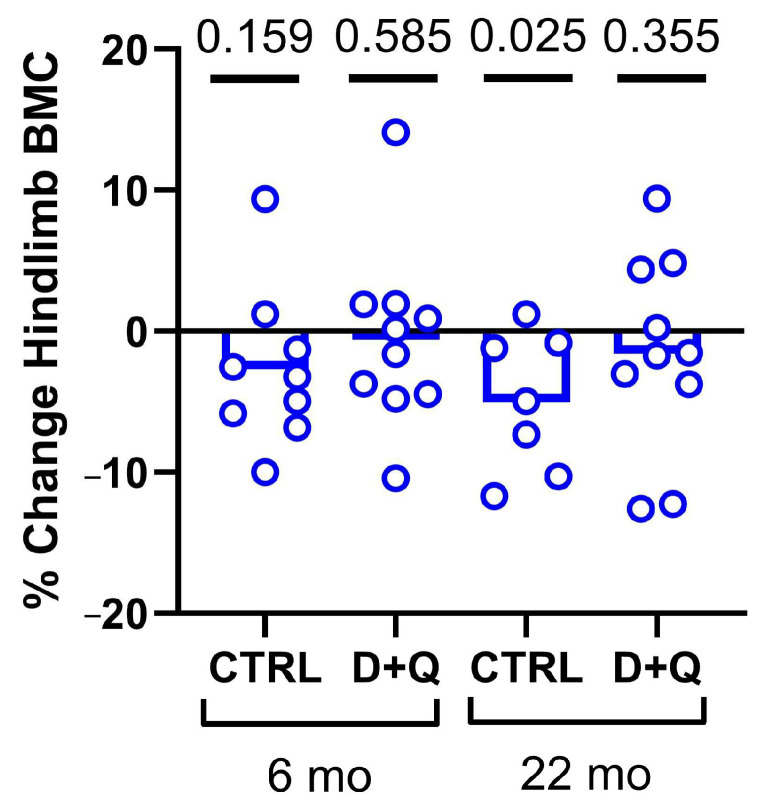
Changes in DXA-derived bone mineral content in the left hindlimb of mice, calculated from before the experiment began (baseline) versus just before sacrifice (endpoint). The 22 mo. mice exhibited bone loss when given the vehicle but preserved BMC when administered intermittent D + Q. Hindlimb analysis consists of bones distal to the acetabulum.

Previous work has shown the significant efficacy of senolytic drug treatment (Dasatinib + Quercetin, hereafter “D + Q”) on the clearance of senescent osteocytes in vivo [2]. To repeat and validate the effectiveness of senolytic therapy for our experimental model, we treated 6-month and 22-month-old WT mice with weekly senolytic (5 mg/kg Dasatinib; 50 mg/kg Quercetin) or vehicle treatment (10% PEG-400) for a total of four weeks, administered via oral gavage. To establish a baseline and end point, we collected DXA scans prior to the first treatment and several days before sacrifice (see Figure 1). The 22 mo. mice given vehicle gavage exhibited a significant reduction (*p* = 0.02) in hind limb bone mineral content (BMC) over the 7-week experimental period. However, the 22 mo. mice given D + Q treatment did not lose bone. No significant changes were detected in the 6 mo. mice, in terms of bone loss, either with or without D + Q treatment (Figure 1). Furthermore, in a separate set of 22 mo. old mice, we assayed the bone tissue for changes in senescence markers after 4 weeks of D + Q treatment. Long bone expression of p15, p16, p21, and p53 were all reduced by D + Q but significance was not reached (Supplementary Figure S1). We also prepared frozen femur sections from these mice for β-Gal staining, but no qualitative difference was detected in among treatment groups. Osteoclast prevalence was also unaffected by D + Q treatment (Supplementary Figure S2). These data, indicating that D + Q protects the aged skeleton from bone loss and (non-significantly) reduces some senescence markers, are consistent with our previous results [40] and suggest that D + Q works in 22 mo. mice.

### 2.2. Treatment with Dasatinib Plus Quercetin (D + Q) Improves the Periosteal Response to Ulnar Loading in Mature Mice

**Figure 2:**
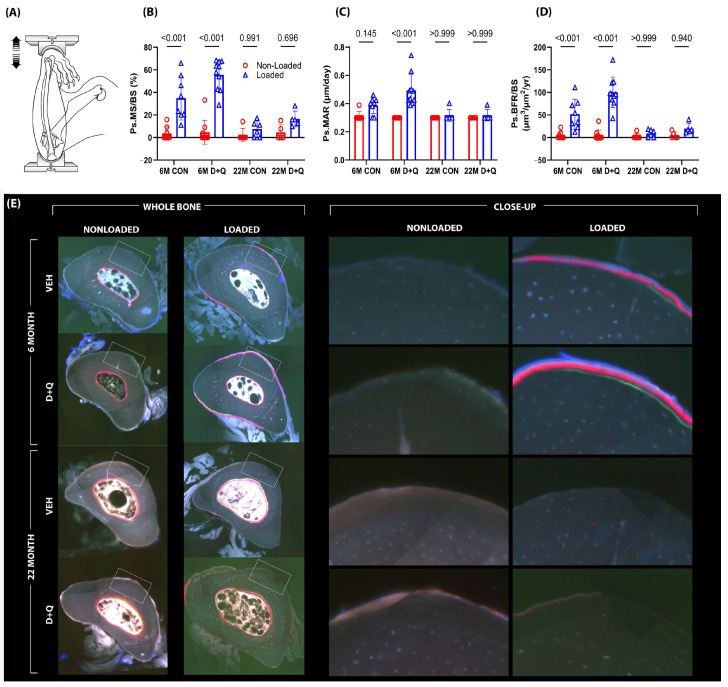
(**A**) Mechanical loading experiments were conducted using the ulnar axial compression model in C57BL/6 mice aged to either 6 months or 22 months. During the 1-month period leading up to mechanical loading sessions, mice were treated with either vehicle or D + Q, once per week for the four week “clearance” period. Bone formation rates were measured using fluorochrome labels injected during the loading experiment (**E**) and quantification was made using standard histomorphometry procedures. The left frame represents the entire bone cross-section analyzed during histomorphometry, while the right frame represents a close-up image of the fluorescent labeling that was measured. (**B**–**D**) The 6-month-old vehicle-treated mice were responsive to loading, but the 6-month D + Q-treated mice exhibited significantly greater responses than the vehicle-treated group. No effects were detected in the 22-month-old mice. Probabilities from 2-way ANOVA, with post-hoc tests, are shown.

To determine whether senescence might impact bone mechanoresponsiveness and implicate a role for senotherapeutic agents in restoring the response, 6-month and 22-month-old mice were given once-weekly treatment with D + Q by gavage, or once-weekly treatment with vehicle by gavage, for 4 weeks. Following the 4-week senolytic treatment period—designed to clear out senescent cells—the mice were subjected to in vivo unilateral ulna loading (see Supplementary Figure S3). As expected, in both 6 mo. and 22 mo. mice, there was negligible bone formation activity in the non-loaded ulna. Among 6 mo. old mice, significant load-induced increases in cortical bone formation parameters (measured from fluorochrome labeling on histological sections) were detected regardless of treatment. However, compared to vehicle-treated mice, the D + Q-treated mice were significantly more responsive to the same mechanical loading protocol, as revealed in paired *t*-tests by a 46% increase (*p* = 0.046) in mineral apposition rate (MAR), a 48% increase (*p* = 0.009) in mineralizing surface per unit bone surface (MS/BS), and a 69% increase (*p* < 0.001) in bone formation rate per unit bone surface (BFR/BS), suggesting that senolytic treatment improves skeletal mechanoresponsiveness at 6 months of age (Figure 2). Similar measurements were carried out on the 22 mo. mice, but no significant changes in quantitative improvements induced by D + Q treatment for mechanically induced ulnar bone formation parameters were observed. It should be noted that when sections from the 22 mo. old mice were being measured, we observed that 100% of the loaded ulnas from the D + Q group exhibited some degree of detectable fluorochrome labeling, whereas only ~65% of the vehicle-treated ulnas had any detectable labeling. This encouraging but muted D + Q periosteal response in older mice prompted us to further investigate senolytic efficacy in a more appropriate loading model (endocortical surface of the loaded tibia) for the aged mouse skeleton (see Section 2.3).

### 2.3. D + Q Treatment Improves the Endocortical Response to Tibial Loading in the Aged Skeleton

**Figure 3:**
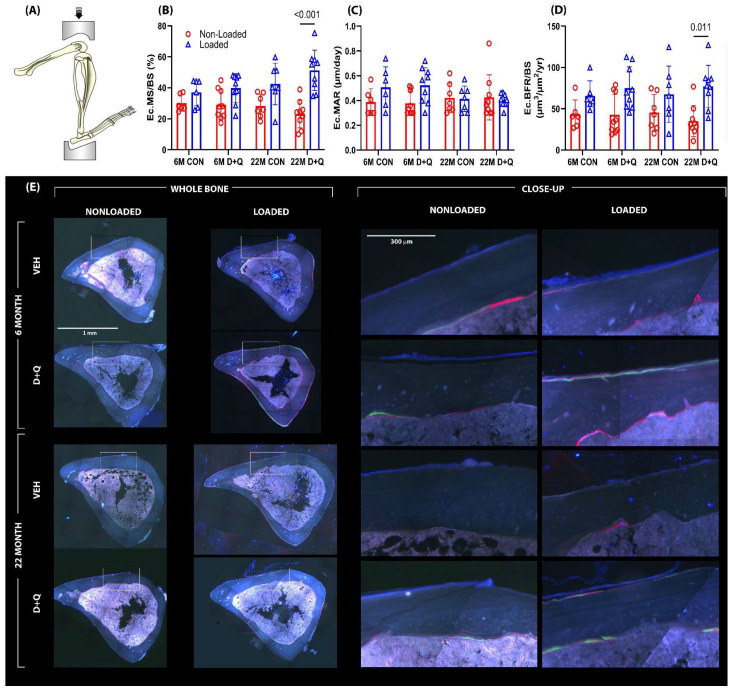
(**A**) Additional loading experiments were conducted utilizing a tibia axial loading model. The tibia axial loading model is a more appropriate model for the aged mice as it is used to evaluate the endocortical surface of the tibia, which is much more active in an aged skeleton and can better represent loading effects consistent with aging. Bone formation rates were measured using fluorochrome labels injected during the loading experiment (**E**) and quantification was made using standard histomorphometry procedures. The left frame represents the entire bone cross-section analyzed during histomorphometry, while the right frame represents a close-up image of the fluorescent labeling that was measured. (**B**–**D**) The 22-month-old mice treated with D + Q had exhibited a significant increase in MS/BS and BFR compared to vehicle-treated mice. Probabilities from 2-way ANOVA, with post-hoc tests, are shown.

The ulnar loading experiments, described in Section 2.2, revealed a D + Q-induced doubling of mechanically induced periosteal bone formation in adult (6 mo.) mice, but aged (22 mo.) mice failed to exhibit quantitative gains in load-induced ulnar periosteal bone formation with senolytic treatment. In retrospect, the lack of responsiveness under any conditions (D + Q or otherwise) in the 22 mo. mice is perhaps not surprising given that a periosteal loading assay was used—the mouse ulna loading model is powerful for assaying periosteal bone formation but is a poor choice for endocortical bone formation [41,42,43,44]. A better model for mechanotransduction in the aged skeleton is to evaluate the endocortical surface of the tibia in the context of the tibia axial loading model. The endocortical surface is more active in the aging skeleton and can reveal loading effects more consistently in rodents of advanced age [45]. To determine if senolytic drug pre-treatment had an effect on mechanically induced endocortical bone formation, a similar cohort of D + Q-treated or control mice (as described above) was subjected to unilateral axial tibial loading. Here, we modified our approach and used peak load magnitudes associated with strains much closer to the osteogenic threshold, so that there was more “osteogenic room” to manifest a potential effect of D + Q on the loading response. Among the 6 mo. mice given vehicle treatment, paired *t*-tests revealed that loading failed to increase endocortical bone formation parameters significantly (Figure 3). However, 6 mo. mice given D + Q exhibited a significant, load-induced increase in endocortical MS/BS (37% load-induced increase; *p =* 0.002) and BFR/BS (52% load-induced increase; *p =* 0.019). Load-induced MAR changes in these D + Q-treated mice failed to reach significance (*p =* 0.066). Among the 22-month-old mice, vehicle treatment was associated with load-induced changes only in MS/BS (50% load-induced increase; *p =* 0.023). The 22 mo. old mice treated with D + Q more than doubled their endocortical MS/BS and BFR/BS in response to loading (122% load-induced increase in MS/BS; *p* < 0.001 and 119% load-increase in BFR/BS; *p* = 0.003; Figure 3). MAR was not improved by D + Q in the loaded limb of the 22 mo. old mice (*p* = 0.9). Load-induced periosteal bone formation parameters were not different between D + Q- and vehicle-treated aged mice. As the proximal tibia metaphyseal region is nearly devoid of cancellous bone by 22 mo. (leaving nothing to measure), we studied the more trabecular-rich epiphyseal cancellous compartment in the proximal tibia for load-induced changes. D + Q treatment significantly improved trabecular BMC and increased BV/TV and Tb.Th. Trab. BV/TV and Tb.Th for D + Q-treated aged mice were at near-significant levels (*p* = 0.054–0.086; Supplementary Figure S4) compared to vehicle-treated aged mice. In summary, D + Q pre-treatment was associated with improved endocortical responses to mechanical stimulation in the aged mouse skeleton, with more muted effects in the adult (6 mo.) skeleton.

### 2.4. Load-Induced Transcription of SASP Genes Is Altered in Mature and Aged Mouse Bone

**Figure 4:**
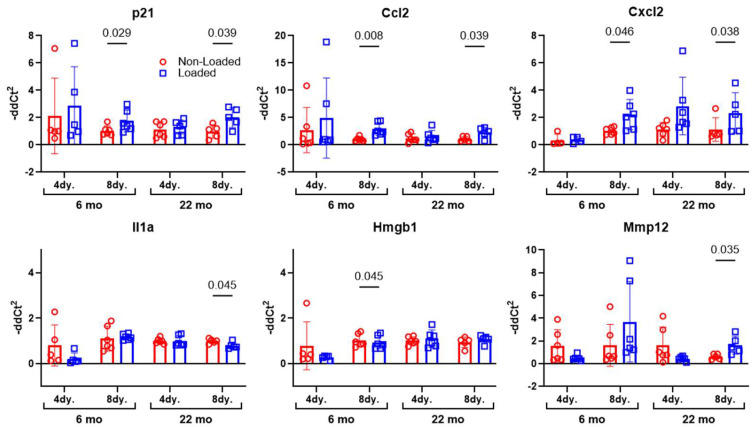
To determine changes in SASP factors with loading, 6- or 22-month-old male mice were subject to a single bout of tibia axial loading. The mice were sacrificed 4- or 8-days post-loading and osteocyte-enriched RNA was isolated from the tibial shafts. Gene expression changes in transcripts associated with known SASP markers were measured and revealed that several SASP genes have a naturally occurring (likely beneficial) role in mechanotransduction signaling within bone. However, as the SASP can be highly cell specific, and the precise composition of the osteocyte SASP (the cell population most represented in these lysates) is not clearly defined, there might be other key osteocyte-selective SASP markers that were not evaluated in our studies.

Senolytic therapy (i.e., D + Q) is not the only senotherapeutic tool available to inhibit the effects of senescent cells on neighboring healthy cells. Inhibition of the SASP, e.g., senomorphics, rather than inducing apoptosis of senescent cells, e.g., senolytics, is another approach. However, normal mechanical signaling in bone involves the release of, and signaling by, a number of inflammatory cytokines (at controlled levels and distribution), many of which overlap with the SASP. Blanket inhibition of these signals from senescent but also healthy cells—as would occur with a senomorphic—might weaken mechanotransduction responses. Tibia mechanical loading experiments were conducted on adult and aged mice and the mice were sacrificed several days later to assay changes in the expression of known SASP factors. The mice underwent tibia loading for one session at equivalent strain magnitudes, as described above. RNA was immediately isolated from the tibial diaphyses and TaqMan-based real-time PCR was performed for a subset of known osteocyte SASP markers, identified in our previous work [15]. p21, Ccl2, Cxcl2, Hmgb1, Il1a, and Mmp12, were among the SASP genes that exhibited changes in expression with loading in either 4-day or 8-day post-loading samples (Figure 4). As these mice are pharmacologically and genetically naïve (i.e., they are normal, WT mice), the naturally occurring increase in several genes that are associated with the SASP (e.g., HMGB1, Il6, and Mmp13) suggest that senomorphic treatment might interfere with mechanical signaling from otherwise healthy cells in bone.

## 3. Discussion

Exercise among seniors is a commonly used strategy to improve manifold health outcomes, including mental health and cognition, metabolic function, cardiovascular health, balance/coordination, sleep, strength, and cancer susceptibility, among others [6,7,18]. Regarding bone health, many seniors engage in exercise to improve skeletal health and reduce skeletal frailty. However, both human clinical studies and animal experiments indicate that skeletal mechanotransduction loses efficacy as the skeleton ages [46,47,48,49,50], making exercise/loading much a much less lucrative activity for improving or maintaining skeletal health compared to earlier decades. It is noteworthy that the decline in load-induced bone formation mirrors the increase in the accumulation of senescent skeletal cells [51], suggesting that there might be a link between senescence-based cellular dysfunction and a loss of mechanotransduction. The main goal of this study was to determine if the clearance of senescent cells, via treatment with a senolytic agent, could improve the load-induced bone formation response in the mature (6-month) and aged (22-month) skeleton. Using two different loading models, we found that a 1-month course of the senolytic cocktail Dasatinib plus Quercetin resulted in improved skeletal response to mechanical stimulation, though in a surface-selective manner with aging.

The results from the ulna loading experiments indicate that D + Q pre-treatment in 6 mo. old mice was able to induce twice as much load-induced bone formation compared to vehicle pre-treatment. This large effect size for D + Q was surprising because 6-month-old mice are reported to have low levels of senescent skeletal cells. One possibility for the efficacy of D + Q in 6-month mice could be related to the recently reported existence of pre-senescent cells [52] on which D + Q might have biological activity. However, we do not have experimental evidence for such an effect. Another possibility is that either Dasatinib, Quercetin, or both have off-target effects beyond the restoration of apoptosis in senescent cells. Lack of periosteal mechanoresponsiveness in the ulnae of 22 mo. old mice given D + Q is disappointing, particularly since periosteal gains translate to disproportionately larger increases in whole bone mechanical properties than equivalent gains on the endocortical surface. However, the more metabolically active endocortical surface in aged mice was sensitive to D + Q-induced improvements in loading effects, as indicated by the results from the axial tibia model. On that surface, D + Q was able to improve the bone formation parameters MS/BS and BFR/BS significantly in the 22-month-old mice, reaching more than twice the gains measured in vehicle-treated 22 mo. old mice. The lack of effect of D + Q on ulnar responsiveness in aged mice, contrasted to the observed effect of D + Q on tibial (endocortical) responsiveness in aged mice, is likely due to the bone envelope or surface selectivity that the two models are designed for. The ulnar model is powerful for assaying periosteal bone formation but is very poor for endocortical bone formation [53]. A more reliable and robust model for mechanotransduction in the aged mouse is to evaluate the endocortical surface of the tibia in the context of the tibia axial loading model [41,44]. The endocortical surface is more active in the aging skeleton and can reveal loading effects more consistently with aging [42,43,45].

The D + Q treatment we used is systemically active, that is, it is accessible to all or nearly all cells. Consequently, it is not possible to determine from our experiments whether clearance of any one particular cell type can be implicated in the improved mechanoresponsiveness. The osteocyte is emerging as a major orchestrator of mechanical effects on bone adaptation, and the process of mechanotransduction in bone relies on a healthy, functional osteocyte population [21,34,35]. Our recent work has shown that osteocytes undergo senescence with increased age [39], so it is tempting to assign the benefits of D + Q treatment to an improvement in the health of the osteocyte population. However, osteoblasts, lining cells, mesenchymal progenitors, or nearby supporting cells (endothelium, sensory nerves, and a variety of cell types in the marrow) might be driving factors in the D + Q effects. Dissection of cell-specific effects will benefit from loading experiments that use genetic mouse models harboring conditional alleles capable of directing senescent cell clearance/apoptosis to specific types and/or populations (e.g., p16^LOX-ATTAC^ recombined with various skeletal-lineage Cre drivers).

We were unable to demonstrate an age-related deficit in several skeletal properties, and complete senescent cell clearance with D + Q was not observed; therefore, these findings should be interpreted as exploratory evidence supporting the potential modulatory role of senolytics in skeletal mechanoadaptation. It will be important to determine whether similar benefits of senolytics on load-induced bone gain are manifest in female mice, as we used only male mice in these experiments. Further, we tested only one dose of D + Q (5 + 50 mg/kg) and one treatment duration (4 weeks). It is possible that alternative dosing regimens would yield more consistent or beneficial results, including the potential to improve the periosteal surface. It is also unknown whether the changes induced by senolytic treatment will have lasting effects on bone mass and architecture induced by mechanical loading. Other bone-building therapies have a limited anabolic window, beyond which bone mass returns to baseline if a subsequent therapeutic (e.g., bisphosphonate) is not used.

We identified a number of SASP factors that are upregulated in mature and aged bone tissue as a result of loading. Bone cell mechanotransduction is known to rely on numerous inflammatory factors, in well-controlled amounts and distribution, in order to achieve proper outcomes [54]. The crucial involvement of inflammatory factors has led many to regard bone cell mechanotransduction as “inflammation on a leash.” Consequently, inhibiting the release of those factors from healthy, non-senescent cells (as occurs with systemic senomorphic administration) might be an inferior approach to reducing the caustic environment, rather than removing senolytic cells and sparing the healthy cells so they can engage in mechano-signaling unencumbered. We did not validate those SASP gene expression changes at the protein level, so the implications of transcriptional responses should be considered cautiously. Focused senomorphic studies that include analysis of protein changes will undoubtedly provide greater insight into the utility of SASP inhibition with loading.

It is unclear which mechanotransduction pathways and signaling mechanisms are altered by D + Q treatment in our loading studies. We and others have reported previously that mechanical stimulation of bone cells induces nitric oxide and prostaglandin release, as well as ERK phosphorylation and P2X7 receptor activation [53,55,56,57]. All of these mechanisms are required for proper mechanical signaling in bone. Of interest, culturing healthy MLO-Y4 osteocyte-like cells with SASP-enriched media collected from senescence-induced MLO-Y4 or MC3T3 cells suppresses all of those mechanisms (PGE2, NO, p-ERK, and P2X7 activity) under fluid flow shear stress conditions [38], suggesting that those pathways might be targeted by D + Q in its enhancement of load-induced bone formation. However, definitive evidence for the precise mechanotransduction pathways altered by senolytics will require a focused and systematic evaluation of each factor.

In summary, the senolytic treatment D + Q was effective in restoring/improving responsiveness to mechanical loading in older (22-month) mice, though the effects were confined to the endocortical surface. Adult (6-month) mice exhibited improved periosteal mechanoresponsiveness from D + Q treatment, presumably by targeting pre-senescent cells. As next generation senolytics are developed and approved, there will be considerable interest in determining whether these agents can improve skeletal outcomes when used in conjunction with moderate exercise protocols.

## 4. Materials and Methods

### 4.1. Mice

For the senolytic loading study, we acquired male C57BL/6 mice at 6 months or 22 months of age from the NIA aging colony. The mice were acclimated to the IU vivarium for one week, then each age group was randomized into one of two treatment groups: vehicle or D + Q treatment. All mouse procedures were performed in accordance with the IACUC guidelines and approvals.

### 4.2. Senolytic Drug Treatment

Mice received once-weekly treatment with D + Q (5 mg/kg Dasatinib; 50 mg/kg Quercetin) by oral gavage or once-weekly treatment with vehicle (10% Polyethylene Glycol-400, PEG-400) by oral gavage, for 4 consecutive weeks. Dasatinib was purchased from Millipore Sigma (Merck, Darmstadt, Germany) and Quercetin was purchased from Santa Cruz (Santa Cruz Biotechnology, Dallas, TX, USA). PEG-400 was purchased from Fisher Scientific (Fisher Scientific, Fair Lawn, NJ, USA). Prior work with senolytics has found that the half-lives for Dasatinib and Quercetin are 4 h and 11 h, respectively [58,59].

### 4.3. Dual-Energy X-Ray Absorptiometry (DXA)

Preliminary and endpoint DXA measurements were collected on the experimental mice on a PIXImus II (GE Lunar) densitometer. The densitometer was calibrated at the beginning of each scanning day using a plastic mouse density phantom provided by the manufacturer (BMD = 0.0611 g/cm^3^; fat = −1.2%). Mice were anesthetized via inhalation of 2.5% isoflurane (IsoFlo, Abbott Laboratories, North Chicago, IL, USA) mixed with O_2_ (1.5 L/min) for a total ~8 min, including both induction and scanning. Mice were oriented in a prone position on the specimen tray within the scanner. Hindlimb scans of the left leg were analyzed using the Lunar region of interest (ROI) tools. The ROI for the hindlimb included all skeletal tissue distal the acetabulum. Scans were performed at the beginning of the study (6 mo. and 22 mo. old) and at the end of study prior to sacrifice (6.5 mo. and 23.5 mo. old). Bone mineral content (BMC) was measured for each ROI scan. DXA endpoints were analyzed with the repeated measures ANOVA using senolytic drug treatment and age as the main effects.

### 4.4. In Vivo Mechanical Loading

The right ulnas and tibias of mice were subjected to in vivo axial tibia or ulnar loading, using the previously described instrumentation. Under isoflurane-induced anesthesia, mice were loaded for 180 cycles/day, 2 Hz, using a haversine waveform. Loading was conducted every other day, for a total of three days (M-W-F). Twelve days after the initiation of mechanical loading, all mice were sacrificed, and bone tissues were collected for plastic-embedded histomorphometry analysis. Ulnar loading was conducted on a customized electromagnetic actuator (TA Instruments ElectroForce Testbench, New Castle, DE), at peak forces of 2.6 N (6 mo.) and 2.45 N (22 mo.), which generates ~2100 µε at the ulna midshaft. Tibial loading was conducted at peak forces of 8.9 N (6 mo.) and 8.6 N (22 mo.), which generates ~1780 µε at the tibia midshaft. Peak loads during mechanical loading were selected based on strain calculations derived from previously published in situ strain gauge analysis by our group [60] and others [61]. Left forearms and tibias were left unloaded and served as internal controls to analyze loading effects on the loaded contralateral side.

### 4.5. Histomorphometry

All mice were given 200 μL intraperitoneal (i.p.) injections of calcein (12 mg/kg) on day 27 of the study (day 0 post mechanical loading) and alizarin complexone (20 mg/kg) on day 34 of study (day 7 post mechanical loading) to periodically label mineralizing bone due to mechanical loading. After sacrifice at day 40 of study, both tibias and ulnas were processed for plastic-embedded histomorphometry and cut at midshaft for histological evaluation. Periosteal and endocortical bone formation rates (BFR/BS; μm^3^/μm^2^/day), mineral apposition rates (MARs; μm/day), and mineralizing surface (MS/BS; %) were calculated from sequential pairs of labels incorporated throughout the experimental period, as described elsewhere [62]. The histomorphometry data were measured and calculated using ImagePro Express (version 6.0, MediaCybernetics, Rockville, MD, USA).

### 4.6. Gene Expression

Mouse ulnas and tibias were collected after 4 or 8 days of a single loading bout, the ends were cut off to allow bone marrow to be removed through centrifugation, and the resulting bone tubes were flash frozen in liquid nitrogen for RNA preparation. The flash-frozen bones were powdered using a mortar and pestle and were immediately mixed with TRIzol (Invitrogen, Thermo Fisher Scientific, Waltham, MA, USA) on ice to maintain RNA integrity and stability. RNA isolation was performed utilizing the PicoPure RNA isolation kit and protocol (Applied Biosystems, Thermo Fisher Scientific, Waltham, MA, USA). Reverse transcription to cDNA was then carried out for qPCR using the High-Capacity RNA to DNA Kit from Applied Biosystems. qPCR was performed on 15 SASP markers identified previously [2], including: p16, p21, p53, Ccl2, Cxcl2, Hmgb1, Igfbp2, IL1a, IL1b, IL6, Mmp9, Mmp12, Nfkb1, Serpine1, and TNFα. Target gene expression was normalized by Hprt1, a housekeeping gene.

### 4.7. Statistics

Statistical analyses were computed with JMP (version 12.0, SAS Institute Inc., Cary, NC, USA) and GraphPad Prism (version 10.0, GraphPad Software, Boston, MA, USA). Paired *t*-tests within-mouse were used to test for loading effects in the various histomorphometric parameters. One-way ANOVA was used to test among groups for DEXA measurements. Statistical significance was taken at *p* < 0.05. Two-tailed distributions were used for all analyses. Data are presented as mean ± SEM. A minimum of eight animals were included in each group (*n* = 8–10/group).

## Data Availability

The original contributions presented in this study are included in the article/Supplementary Materials. Further inquiries can be directed to the corresponding author.

## Supplementary Materials

The following supporting information can be downloaded at: https://www.mdpi.com/article/10.3390/ijms262211233/s1.
