# Supplementary material for: Senolytic Treatment Improves Responsiveness to Mechanical Loading in the Skeleton of Aged Mice
Source: Int J Mol Sci. 2025 Nov 20;26(22):11233. doi: 10.3390/ijms262211233 (PMC12653123; doi:10.3390/ijms262211233)
Supplement: Supplementary file 1 [file ijms-26-11233-s001.zip › ijms-3792357-supplementary.pdf]

Supplemental Materials

Supplemental Figure S1

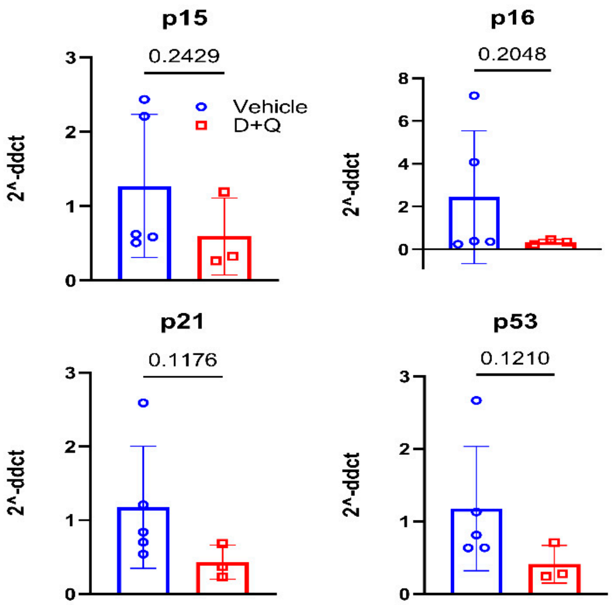

Supplemental Figure S1: Graphical illustration of qPCR senescent markers measured from pulverized frozen humerus samples and normalized to housekeeper gene, GAPDH. p15, p16, p21, and p53 were all analyzed in 22-month mice to determine changes in expression due to treatment with D+Q senolytic treatment. All 4 senescence markers observed a decrease in expression from vehicle to D+Q treated mice, though none of the changes were statistically significant ($0.11<p<0.24$).

Supplemental Figure S2

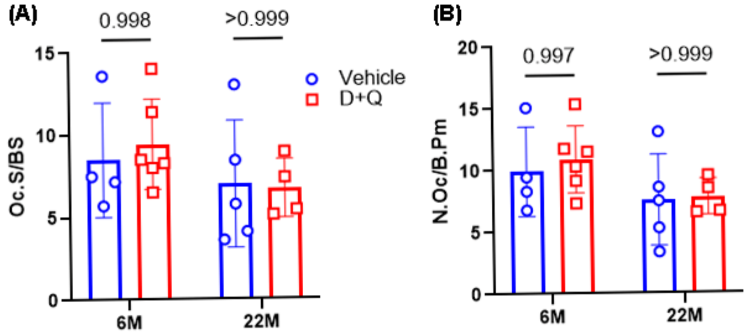

Supplemental Figure S2: Graphical illustration of osteoclast TRAP staining quantification of proximal tibiae sections. No significant difference was observed between vehicle and D+Q treated mice in either age group in both **(A)** osteoclast number per total area of bone surface and **(B)** osteoclast number per bone perimeter analyzed.

Supplemental Figure S3

				EXPERIMENTAL DAY																																						
Age	n=	Treatment		1	2	3	4	5	6	7	8	9	10	11	12	13	14	15	16	17	18	19	20	21	22	23	24	25	26	27	28	29	30	31	32	33	34	35	36	37	38	39
22 mo	9	Senolytic		Dxa	D+Q							D+Q							D+Q						D+Q	Load		Load		Load Calcein							Aliz.			Dxa		Sac
22 mo	9	Control		Dxa	Veh							Veh							Veh						Veh	Load		Load		Load Calcein							Aliz.			Dxa		Sac
6 mo	8	Senolytic		Dxa	D+Q							D+Q							D+Q						D+Q	Load		Load		Load Calcein							Aliz.			Dxa		Sac
6 mo	8	Control		Dxa	Veh							Veh							Veh						Veh	Load		Load		Load Calcein							Aliz.			Dxa		Sac

Supplemental Figure S3: Graphical illustration of the experimental design used to investigate the effects of senolytic treatment on mechanically induced bone formation in adult (6 month) and aged (22 month) mice. Mice were treated for 1 month with either senolytic or vehicle treatment, loaded for 1 week, exposed to fluorochrome labels (calcein and alizarin) to monitor new bone formation, then sacrificed. DXA scans were collected prior to the start of treatment and prior to sacrifice. DXA = dual-energy x-ray absorptiometry; D+Q = Dasatinib (5 mg/kg) + Quercetin (50 mg/kg) combination drug treatment session; Veh = 10% PEG-400 vehicle drug treatment session; Load = single, 3 minute ulnar and tibial loading session; Calcein = 200μL intraperitoneal injection of calcein; Aliz = 200μL intraperitoneal injection of alizarin complexone; Sac = sacrifice of animal and collection of bone tissue for analysis.

Supplemental Figure S4

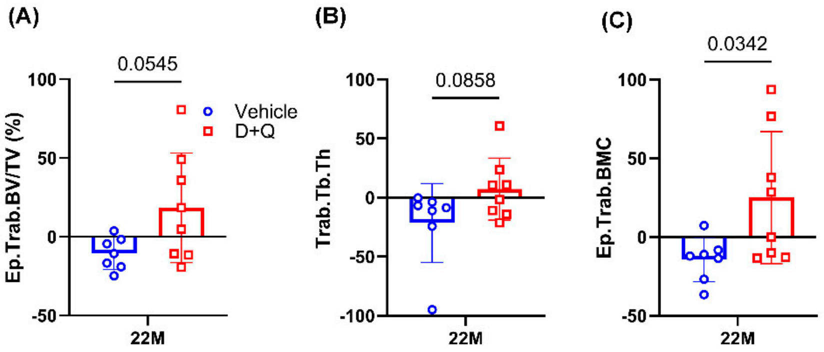

Supplemental Figure S4: Graphical illustration of relative (intra-animal percent change for loaded vs unloaded) changes in proximal tibia epiphyseal **(A)** bone volume fraction (BV/TV); **(B)** trabecular thickness (Tb.Th); and **(C)** trabecular bone mineral content (BMC) induced by loading, among 22 mo. mice.

Supplemental Methods

We conducted power analysis to determine sample sizes needed for estimated changes in load-induced bone formation induced by senolytic treatment. We used previously published data (Nat Med 2017, Figure 2) addressing the effect of D+Q treatment on endocortical bone formation rates to estimate the effect size of D+Q treatment. Here, we considered an increase of Ec.BFR from 0.48 in vehicle treated to 0.80 in D+Q treated to calculate sample sizes for the current study. Given that estimated effect size, and specifying 80% power at α=0.05, we calculated that a minimum sample size of n=6 was necessary to find a significant D+Q effect on loading, if one existed. Therefore, all of our group sizes have a minimum of n=6.
